# Evolution of protein complexes by duplication of homomeric interactions

**DOI:** 10.1186/gb-2007-8-4-r51

**Published:** 2007-04-05

**Authors:** Jose B Pereira-Leal, Emmanuel D Levy, Christel Kamp, Sarah A Teichmann

**Affiliations:** 1 Instituto Gulbenkian de Ciência, Apartado 14, P-2781-901 Oeiras, Portugal; 2 MRC Laboratory of Molecular Biology, Hills Road, Cambridge CB2 2QH, UK; 3 Paul-Ehrlich-Institut, Federal Agency for Sera and Vaccines, Paul-Ehrlich-Straße, 63225 Langen, Germany

## Abstract

**Background:**

Cellular functions are accomplished by the concerted actions of functional modules. The mechanisms driving the emergence and evolution of these modules are still unclear. Here we investigate the evolutionary origins of protein complexes, modules in physical protein-protein interaction networks.

**Results:**

We studied protein complexes in *Saccharomyces cerevisiae*, complexes of known three-dimensional structure in the Protein Data Bank and clusters of pairwise protein interactions in the networks of several organisms. We found that duplication of homomeric interactions, a large class of protein interactions, frequently results in the formation of complexes of paralogous proteins. This route is a common mechanism for the evolution of complexes and clusters of protein interactions. Our conclusions are further confirmed by theoretical modelling of network evolution. We propose reasons for why this is favourable in terms of structure and function of protein complexes.

**Conclusion:**

Our study provides the first insight into the evolution of functional modularity in protein-protein interaction networks, and the origins of a large class of protein complexes.

## Abstract 2

A study of yeast protein complexes, complexes of known three-dimensional structure in the Protein Data Bank and clusters of pair-wise protein interactions in the networks of several organisms revealed that duplication of homomeric interactions often results in the formation of complexes of paralogous proteins.

## Background

The success of genome sequencing projects has resulted in the accumulation of catalogues of genes for hundreds of genomes. Within each genome, the genes and their proteins interact to form complex networks with properties that transcend those of individual genes. One such network is formed by the totality of physical protein-protein interactions in the cell: the protein interaction network (PIN). These networks, like many other naturally occurring networks, such as the transcriptional [1,2] and metabolic networks [3], have a modular organization [4-6]. They are organized into a number of functional modules, which are sets of interacting proteins accomplishing discrete biological functions in relative spatial, temporal or chemical isolation from other modules in the network [6]. Protein complexes are functional modules in the sense that the protein subunits of the complex are sufficient for its function, even when isolated from the system, as has been demonstrated by *in vitro *reconstitution of active protein complexes in a variety of studies (for example, [7]).

**Figure 1:**
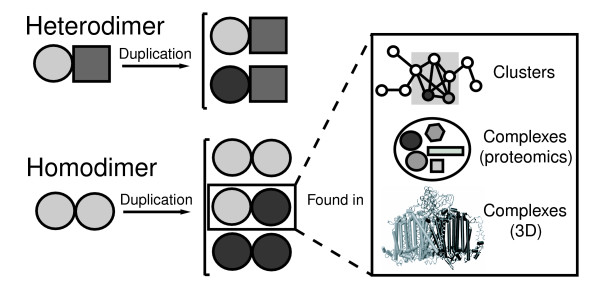
A hypothesis for the origins and evolution of protein complexes. Gene duplication with conservation of protein interactions is frequent [9]. Self-interactions (homomeric interactions) have special structural properties (see text for details) that are conserved into the duplicated interaction between paralogous proteins (light-dark interaction). Interactions between paralogous proteins are more versatile functionally and structurally, and are systematically selected for in evolution. These interactions are central in the establishment and evolution of clusters in PINs and protein complexes.

The mechanisms that drive the emergence and subsequent evolution of modularity in cellular networks are unclear. This is in part due to the fuzzy nature of the concept and the difficulty in identifying functional modules in cellular networks. Here, we focus on the evolution of one specific type of functional module, protein complexes, and propose an evolutionary route that accounts for the origin and evolution of a proportion of these modules. We hypothesize that duplication of self-interacting proteins (homomers) is critical for the establishment and evolution of a proportion of protein complexes, and hence of functional modularity in protein interaction networks (Figure 1). Our hypothesis was based on the following considerations.

First, gene duplication and divergence is the most important force driving the expansion of eukaryotic proteomes (for example, [8]). Conservation of protein interactions is frequent after duplication and paralogous genes thus frequently share interaction partners [9]. Mathematical models of network evolution based on this principle of duplication and divergence result in networks that display topological properties comparable to those of biological protein interaction networks, in particular high clustering coefficients [10,11]. Clusters in protein networks are frequently part of protein complexes [4,12,13]. The clustering coefficient of a network (*C*) is a measure that quantifies how interconnected the proteins are [14], partly reflecting modularity of the network. So duplication followed by conservation of protein interactions is linked with modularity in theoretical simulations of network evolution.

A second piece of evidence is that the oligomerization of multiple, identical subunits is a simple way of forming large, functional structures in a genetically economical manner. Smaller component subunits will fold more readily than a single large protein and are less prone to translational errors [15,16]. Multiple copies of the same protein will tend to be co-localized in the cell as they can be synthesized from the same mRNA. This may promote oligomerization, for example, by domain swapping [17] or other mechanisms. Furthermore, evolution of a homomeric interface by incremental mutation is aided by the fact that the effect of one advantageous mutation will apply to all subunits of a homo-oligomer, and is thus, *a priori*, the most likely type of interface to occur [18]. In protein interaction networks, homomeric interactions are indeed over-represented [19]. They are also very abundant in complexes of known three-dimensional structure, present in 85% of all complexes in the Protein Quaternary Structure (PQS) database (Table 1).

The third consideration is that when genes coding for proteins that form homodimers duplicate, conservation of interactions will generate dimers of paralogous proteins. In these, the stability associated with the homodimer is maintained, while at the same time asymmetry is introduced into the interaction. This asymmetry provides more degrees of evolutionary freedom and represents a source of functional novelty (discussed in [20]). This is illustrated by the anecdotal examples like the photosystem I (Figure 1), in which there is asymmetry in terms of the subunits bound to PsaA and PsaB, the two paralogous proteins at its core [21,22].

These considerations suggest the following evolutionary scenario (see Figure 1), which we test in the work presented here. An initial interaction is established between two (or more) copies of the same protein (homomeric interactions; Figure 1, left). This is the stable 'seed' of a new complex, and functional and structural factors will contribute to this interaction being selected for conservation. Gene duplication and divergence with conservation of the interactions will then follow. This initially results in multiple homomeric and heteromeric complexes with different numbers of the two duplicates (Figure 1, middle), permitting functional and structural diversification. Over time, sequence divergence will produce distinct complexes with distinct functionalities. The complexes containing paralogous proteins will frequently be selected in evolution due to the advantages of asymmetry, and accretion of new interactions may follow. This evolutionary process is illustrated by the related complexes of the RecA recombinase homohexamer and the F1 ATP synthase α3:β3 hexamer (discussed below). These two functionally distinct complexes are likely to have evolved from a common homomeric ancestor [23].

## Results

We test the evolutionary scenario hypothesized above by investigating the following corollaries: whether duplication of genes coding for homodimers is frequently accompanied by conservation of protein interactions in protein interaction networks; whether interactions between paralogous proteins are associated with high clustering in protein interaction networks; whether these interactions are over-represented in protein complexes obtained in large-scale proteomic experiments; whether interactions between paralogous proteins are over-represented in protein complexes of known three-dimensional structure; whether these interactions are older than other interactions and, hence, paralogous dimerization precedes accretion of further interactions, as well as whether the establishment of dimers of paralogues is associated with asymmetry of protein interactions.

### Duplication of homodimers with conservation of interactions

**Figure 2:**
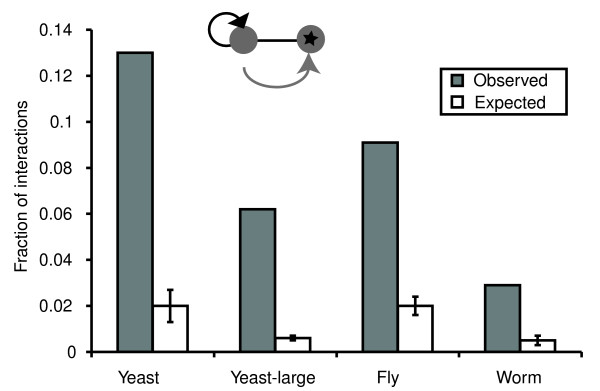
Dimers of paralogues in the protein-protein interaction network. On top is a cartoon illustrating how paralogous dimers result from the duplication of proteins that form homodimers. The bar chart shows the fraction of paralogous dimers (gray bars) in four protein interaction networks compared with random expectation levels, obtained by 10,000 network randomizations by shuffling evolutionary relationships (*p *< 10^-4^; see Materials and methods for details).

It is known from previous work that gene duplication accompanied by conservation of interactions is common in PINs for both homomers and interactions between non-homologous proteins [9,19]. We have calculated the frequency of interactions between paralogues in four independent datasets of protein interaction networks studied here (Table 1). We used structural assignments to detect homology, thus considering even very distant evolutionary relationships, as described in Materials and methods. We found that interactions between paralogues are significantly more frequent than expected by chance (Figure 2). In order to investigate the evolutionary origins of interactions between paralogues, we determined the conditional probabilities for a protein that forms a paralogous dimer to also be a homodimer. The observation that interactions in homodimers and paralogous dimers are not independent (Table 2) supports an evolutionary link between these two types of dimers, such that paralogous dimers evolved by duplication of homodimers. These observations support the corollary that duplication of homomers is frequently accompanied by conservation of interactions.

### Duplication of homodimers and network clusters

**Figure 3:**
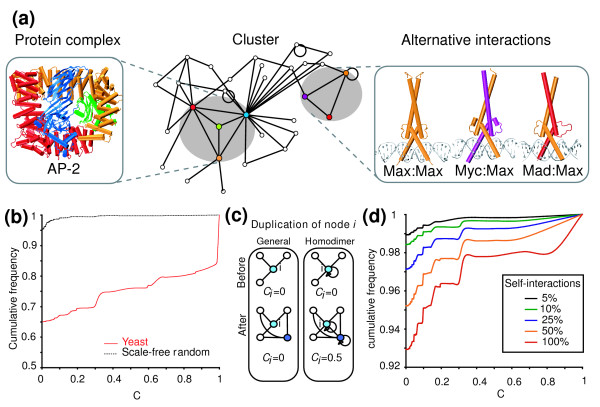
Clusters in PINs. **(a) **A small section of a PIN in *S. cerevisiae *is represented as a graph where nodes correspond to proteins and edges to physical interactions between pairs of proteins. One definition of a module in this work is a highly connected subgraph, such as that shaded in the figure (left), in which the central (green) node has a maximum clustering coefficient (*C *= 1). A clustering coefficient can be calculated for each protein in the network and measures the number of interactions between neighbors of that protein, divided by the total number of possible interactions between those neighbors. In this example, the green node and its fully connected neighborhood correspond to the protein complex AP-2 [49]. Fully connected subgraphs can also represent interactions that are dissociated in time and/or in space. For example, the shaded cluster on the right represents members of the basic helix-loop-helix transcriptional regulator family, in which duplication of a homodimeric protein with inheritance of interactions resulted in Max existing as a homodimer, as well as distinct dimers of paralogous proteins (c-Myc and Mad1) [34,35]. **(b) **Cumulative frequency distribution of the clustering coefficients in the Yeast PIN and in randomized networks with exactly the same degree distribution (scale-free random; see the Randomization by link shuffling section in Materials and methods for details). This shows that high clustering of real PINs, and thus their modularity, is a characteristic of their biology and not of the degree distribution. **(c) **Cartoon illustrating the consequences of duplication with conservation of interactions for the clustering coefficient of node (protein) *i *(*C*_*i*_). In each case the network is shown before and after duplication of a protein that either interacts with itself or does not. The bottom part of the cartoon summarizes the effect on the clustering coefficient of the protein. **(d) **Cumulative frequency distribution of clustering coefficients in the simulated networks, with varying proportions of self-interactors at the start of the simulation. The fraction of proteins with higher clustering coefficients increases with the proportion of self-interactors.

We next investigated whether the duplication of homodimers is associated with protein complexes in PINs. We consider the average clustering coefficient (*C*) of a network as a descriptor of the extent of modularity of that network. Clusters frequently correspond to known protein complexes, as shown by [4,12,13] and by us (Supplementary material S1 in Additional data file 1; illustrated in Figure 3a). This parameter captures the frequency of densely connected subgraphs in the network [14], and allows us to measure modularity in a PIN without specific knowledge of the identity of the protein complexes. We show here that PINs are more clustered than randomized networks with exactly the same broad degree distributions as the real yeast PIN (Figure 3b), extending previous analysis where it was shown that protein interaction networks were more clustered than random Erdõs-Rényi networks and random power-law networks [24]. This provides strong support for the biological significance of clusters in networks.

We investigated whether duplication plays a role in determining the clustering levels of the network. The duplication and conservation scenarios in Figure 3c suggest that only duplication of proteins that form homodimers, and not other proteins, will lead to an increase in the clustering coefficient of the network. To investigate this, we implemented a theoretical model of network growth by duplication-divergence [11,25,26] and asked whether inclusion of self-interactions in the model increases the global clustering coefficient.

As shown in Figure 3d, the presence of self-interacting proteins increases the clustering of the network in this model. In particular, the higher the initial proportion of self-interactors, the higher the clustering of the resulting networks (see Materials and methods and Supplementary material S2 in Additional data file 1 for details of the modeling procedure). This is consistent with the result obtained in a previous theoretical study of network evolution by duplication-divergence [10]. The increases in clustering levels in this simplified model are modest, suggesting that additional mechanisms must operate in the evolution of real networks, and that only a subset of protein complexes are derived by the mechanism proposed here.

Conversely, when we consider the four real PINs (Table 1) and ask the opposite question, whether selective removal of interactions between paralogous proteins reduces the global clustering of the network, we find that this is the case. Clustering levels are reduced by between 7% and 15% (Supplementary material S3 in Additional data file 1). This is significantly more than obtained by removal of other interactions, which has negligible effects on the global clustering of the network. These small but significant reductions are consistent with the modeling results, further supporting that this mechanism operates in the evolution of a subset of protein complexes.

This result is subject to the following caveats. First, in some cases the formation of a cluster is not due to a single multi-protein complex, but many alternative ones, which may not co-exist in time and in space. This has been described in transcription factor families [27,28], and is illustrated in Figure 3a. Secondly, the graph representation we use for PINs is, in itself, limited; for example, it ignores the stoichiometry of the different subunits within protein complexes. For example, a protein complex composed of six identical subunits forming a ring would be depicted as a single self-interacting node, and not captured as a cluster in the PIN. Thus, although considering PINs gives us a network perspective on protein complexes and also large numbers of interactions and increased statistical power, we need to consider alternative definitions of protein complexes to substantiate the above result. So, we next investigated experimentally derived protein complexes.

### Paralogous subunits in protein complexes

We tested the corollary that there is an over-representation of interactions between paralogous proteins within protein complexes. We considered two distinct types of protein complex data. The first is composed of three independent data sets of protein complexes in *S. cerevisiae *(Table 1) and is discussed in this section; the second is composed of protein complexes of known three-dimensional structure, and will be considered in the next section.

**Figure 4:**
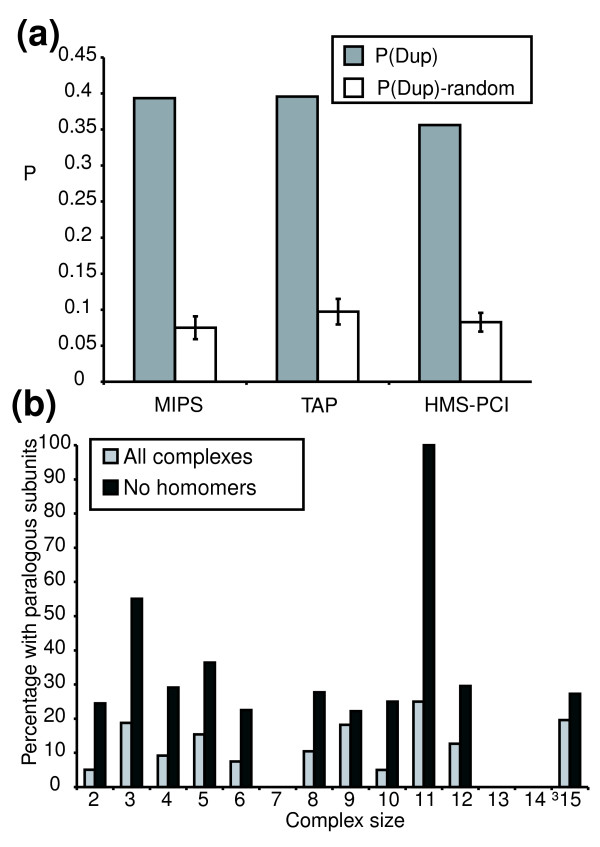
Duplication of subunits in protein complexes. **(a) **Nearly 40% of the protein complexes have homologous subunits (gray bars). These levels are higher than expected by chance (white bars). Random expectation levels are the averages of 10,000 randomized protein complex datasets, where the complex size distribution is kept constant. While the MIPS dataset is the result of manual curation (see table 1), both TAP and HMS-PCI are the result of large-scale experiments, and some redundancy may exist from multiple baits picking up the same complex. For the TAP dataset, the authors provide a smaller set of predicted complexes based on bioinformatics methods. We repeated the calculation on this set of predicted TAP complexes and found that 47% of the complexes have duplicated subunits, while 18 ± 2% would be expected at random. The significance level remains the same for this predicted set of complexes as for the raw purification data. **(b) **Percentage of complexes of known three-dimensional structure that have duplicated subunits, as a function of complex size. Grey bars are for the complete data set, whereas black bars are from a dataset that excludes purely homomeric complexes, as these dominate the dataset (see Table 1) and may distort the results. On average, between 9% and 30% of the complexes display duplicated subunits (including and excluding purely homomeric complexes, respectively). This is not an artifact introduced by the complex size distribution.

First, we found that in all three *S. cerevisiae *datasets, about one-third of the protein complexes contain duplicated proteins, which is more than expected by chance (Figure 4a). We then wanted to check whether the duplicates physically contact each other within these complexes. However, the three *S. cerevisiae *protein complex datasets lack information on the physical interactions, or interfaces, formed between the constituent proteins of a complex, as well as the stoichiometry of the complexes, that is, how many copies of each protein are present. Therefore, we computationally overlaid all the protein interactions (Yeast and Yeast-large) onto the protein complexes and asked whether the paralogous subunits of complexes physically interact. Of the complexes for which protein interactions can be superimposed, 27% to 30% have interacting homologous proteins (Table 1). The TAP and HMS-PCI datasets are the result of large-scale experiments, and some redundancy may exist, deriving from multiple baits picking up the same complex. For the TAP dataset, the authors provide a smaller set of predicted complexes based on bioinformatics methods. We repeated the calculation on this set of predicted TAP complexes and found that the frequencies at which the complexes contain homodimers (F(HD)) or dimers of paralogous proteins (F(PD)) are 43% and 34%, respectively. In all cases we found that this enrichment is highly significant at *p *< 10^-4^.

Thus, analysis of the three sets of *S. cerevisiae *protein complex datasets supports the corollary that interacting paralogues are over-represented amongst protein complexes.

### Paralogous subunits in protein complexes of known 3D structure

Next we concentrated on the set of protein complexes with known three- dimensional structure (Table 1) to further test the corollary that there is an over-representation of interfaces between paralogues within each protein complex. This dataset, obtained from the PQS database, is an automatically generated subset of the PDB containing solely oligomers [29]. In PQS, the proportion of complexes with paralogues is comparable to the *S. cerevisiae *complex datasets, at 30% (Figure 4b). The advantage of studying this dataset is that it can provide stoichiometry and interaction maps for complexes, that is, we can test directly whether paralogues interact.

**Figure 5:**
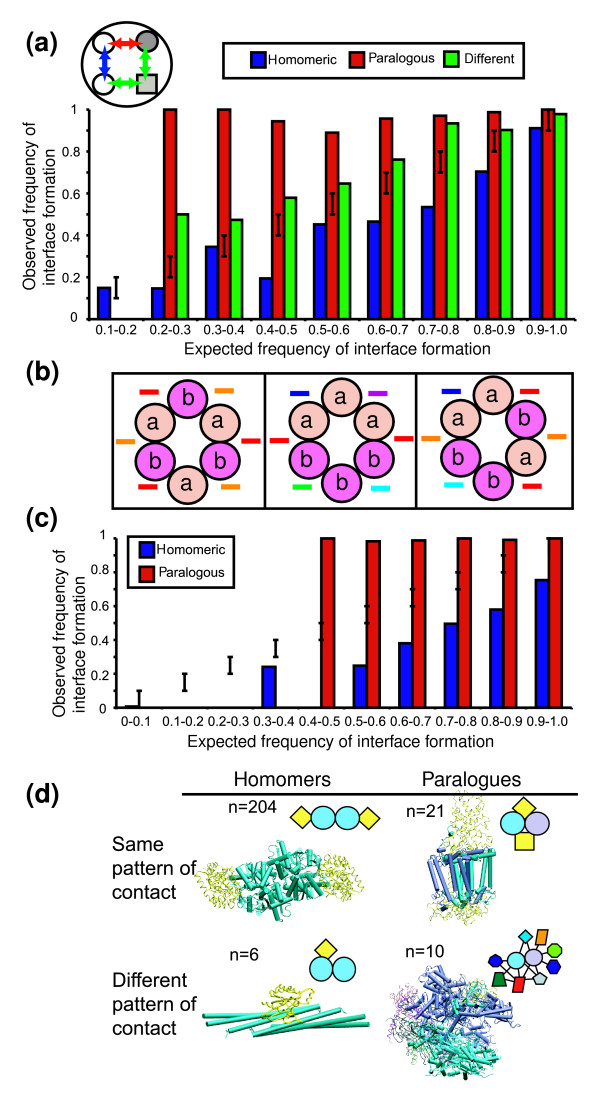
Duplicated subunits in complexes interact. **(a) **Interactions between paralogous subunits (red) are more frequent than expected given the stoichiometry of subunits within protein complexes. Chains from PQS complexes were binned according to probability of forming a homomeric interaction or interactions between paralogous or different chains (see Materials and methods). The frequencies at which these chains form homodimers and paralogous dimers (averaged for each bin) are shown as blue and red bars, respectively. In a random scenario, all the points lie within the range shown in the black lines. **(b) **Possible arrangements of two distinct subunits in a hexameric ring like that of the F1 complex. The actual F1 complex is shown on the left. Bars of different colors correspond to different inter-subunit interfaces. **(c) **If there are multiple identical and paralogous chains within a protein complex, the chains tend to be arranged in three-dimensional space so that the paralogous chains rather than identical chains are contacting each other, corresponding to the scenario shown on the left. Note that when there is a choice, interactions between paralogous proteins are always preferred. This experiment is similar to that described in (a), but considering only the two types of interaction in the calculations. **(d) **The role of oligomers of paralogues in generating structural diversity. n is the number of protein complexes found in PQS that have identical chains (left) or paralogous chains (right), which contact the same (top) or distinct binding partners (bottom). Hetero-oligomers that contain paralogous dimers are more frequently asymmetrical (10/31) than those containing homomers (6/210), that is, paralogues tend to bind different partners. The complexes shown illustrate the four possible situations. Top left is the tryptophan synthase from *Salmonella typhimurium*, in which the homomeric α:α dimer (blue) binds one β subunit on each side (yellow) [50], which represents symmetry in binding partners of homomers. Top right is the photosynthetic reaction centre from *Rhodopseudomonas viridis*, in which both paralogous L and M chains (blue and purple) bind to the H and C subunits (shown in yellow) [51], which illustrates symmetry in the binding partners of a dimer of paralogues. Bottom left is the structure of the Rac1 small GTPase bound to the arfaptin-1 homodimer [52] from *Homo sapiens*, in which Rac1 binds solely one of the arfaptin chains, but occupies a volume that excludes the possibility of additional Rab molecules binding the other arfaptin chain; this illustrates the rare cases of asymmetry in the binding partners of homomers. Bottom right is the RNA polymerase from *S. cerevisiae *[53], in which many peripheral subunits decorate the central core formed by the dimer of paralogues A:B, which illustrates the creation of asymmetry by the duplication of the ancestral homodimer [32].

Consistent with our hypothesis, we found that the frequency of interactions between paralogues within a protein complex is higher than would be expected by chance, while that of homomeric interactions is lower (Figure 5a; see Materials and methods for an explanation on how the expected values are calculated). One example is the mitochondrial F1/Fo ATP synthase complex (Figure 5b), which contains interacting paralogous subunits [30]. While it could potentially establish homomeric contacts, no such contacts exist in the complex, illustrating how homomeric interactions can be under-represented compared to the random scenario. Thus, we have shown that paralogues not only frequently interact within protein complexes, but also appear to interact preferentially compared to homomeric interactions and interactions between evolutionarily unrelated proteins.

To further investigate this, we repeated the experiment shown in Figure 5a, but considering only subunits that can establish homo-interactions as well as interactions between paralogues. This is equivalent to determining what choice is made in a situation such as that represented in Figure 5b. We found that given a choice, in almost all cases a preference for interactions between paralogues will be made, as shown in Figure 5c. The reason for this is likely to be that this type of geometrical arrangement of proteins within complexes requires the smallest number of different interfaces to be formed, and so is the most parsimonious evolutionary scenario. In the F1 sub-complex, the three α and three β subunits alternate within the hexameric ring [30], so that only two different interfaces are formed (α:β and β:α; Figure 5b, left).

### Evolutionary cores of protein complexes and asymmetry

Our hypothesis is that many protein complexes start with homomeric interactions that duplicate and diversify, and serve as a seed for the coalescence of further subunits. The photosystem I shown in Figure 1 illustrates this concept. In *Heliobacteria*, the complex contains a homodimer at its core (PshA_2_), whereas the eukaryotic complex contains a dimer of paralogues (PsaA:PsaB). These two paralogous polypeptide chains are each decorated by different peripheral subunits, suggesting that in this class of photosystem (Type-I RC), the core was established prior to the accretion of further subunits [21,22]. Another example is RNA polymerase II, which contains at its core a large dimer of homologous subunits, and is believed to have evolved from an ancestral generic nucleic acid binding homodimer [31,32].

To investigate whether this is a frequent mechanism of evolution of complexes, we tested the fifth corollary and asked whether homomeric interactions and interactions between homologous proteins precede interactions between unrelated proteins in evolution. Then we tested whether paralogues within complexes of known three-dimensional structure have asymmetric interactions.

To answer the first question, we compared PINs in different organisms and asked whether homomeric interactions and interactions between paralogues in one organism are likely to be conserved in the PINs in other organisms, that is, whether the protein(s) have orthologues that interact. Such pairs of interactions have been termed interologs in [33]. In Table 3 we show that self-interactions and dimers of paralogues are three to seven times more likely to be conserved from yeast to fly and worm than interactions between unrelated proteins. However, due to the small number of conserved interactions, these results are not definitive. To gain a larger coverage of the yeast proteins, we tested whether proteins that establish interactions with identical and/or with homologous proteins are older than other proteins. We estimated the likely time of origin of each gene in *S. cerevisiae *by phylogenetic profiling and analyzed both the Yeast and Yeast-large PINs as described in Materials and methods. Homomeric proteins and those that interact with paralogues are significantly older than other proteins, tending to be present in all Eukaryotes and either in Bacteria or Archaea (Yeast) or all Eukaryota (Yeast-large). Other proteins tend to be present only in Fungi and Metazoa, but not in other Eukaryota. The difference in evolutionary conservation is statistically significant in both data sets (*p *= 0.003 for yeast and *p *< 0.001 for Yeast-large, Wilcoxon-Mann-Whitney test). These two results support the corollary that the establishment of homomeric interactions and the duplication to dimers of paralogous chains are early events in the emergence of a protein complex.

To test whether paralogous proteins break the symmetry of a complex and allow accretion of different types of subunits, we considered the protein complexes of known three-dimensional structure again. We compared the set of complexes that contain paralogues to the complexes that contain homomeric interactions and no paralogues. As shown in Figure 5d, we found that 32% of paralogues have asymmetrical interactions, while only 4% of the homomers do, a significant difference (*p *< 0.001). Thus, the hypothesis that duplication of homomers results in new asymmetrical complexes is supported by the data. This may represent part of the selective advantage for conservation of such duplications.

## Discussion

We present here a genome-wide, cross-species analysis of the origins and evolution of protein complexes. At the beginning, we hypothesized that duplication of self-interacting proteins (homomers) is an evolutionary path leading to the establishment and evolution of many complexes. To substantiate this hypothesis, we tested five corollaries that arise from such an evolutionary scenario.

The first corollary is that duplication of genes coding for homodimers is frequently accompanied by conservation of protein interactions. Conservation of protein interactions after gene duplication has been shown to be frequent [9,19]. We show here that between 4% and 13% of interactions in PINs are between paralogous proteins.

Next we tested the association between clustering of the network and interactions between paralogous proteins. Clusters in protein interaction networks frequently represent protein complexes. We have shown that removal of interactions between paralogues causes a small but highly significant decrease in the global clustering level of the network. This is consistent with our theoretical modeling results.

We then observed that about 30% of protein complexes from proteomics experiments contain duplicated subunits. In protein complexes of known three-dimensional structure, a similar proportion of complexes have duplicated subunits, and more importantly, there is preferential binding of paralogous subunits. This supports the corollary that interactions between paralogues are frequent in complexes.

We observed that proteins involved in homomeric interactions and interactions between paralogues were more conserved than other proteins: more than half of yeast proteins had orthologues in all eukaryotes and either archaea or bacteria, whereas more than half of the other yeast proteins had orthologues only in fungi and animals. Homomeric interactions and those between paralogous proteins were also three to seven times more likely to be conserved, when compared to other interactions. Thus, this supports the corollary that homomers and oligomers of paralogues represent the first steps in the evolution of new protein complexes, with other subunits added later.

Finally, we showed that amongst three-dimensional structures of complexes, 32% of dimers of paralogues establish asymmetric interactions with other proteins whereas only 3% of homodimers show such asymmetry, further substantiating that the duplication of homomeric interactions helps to create asymmetry in protein interactions, and allows the coalescence of other subunits in the complex.

Altogether, our data suggest an evolutionary route to the formation and specialization of many extant protein complexes. On this route, homomers and oligomers of paralogous subunits represent an ancestral core around which further subunits can coalesce in evolution. Sequence divergence of the paralogous subunits creates the asymmetry that permits the accretion and diversification of interactions. In addition, divergence of paralogues may be involved in functional specialization of complexes. The biases inherent in each data type make it difficult to determine the exact fraction of protein complexes that evolved via the proposed route. A higher bound is about one-third, estimated by the fraction of proteomics complexes that display duplicated subunits. A lower bound is less than one-tenth, estimated by the fraction of dimers of paralogues in one of the yeast two-hybrid data sets (Table 1).

Another issue that at this stage is difficult to ascertain is the nature of the complexes that emerged by the proposed route. If we assume that both the proteomics data and the crystallographic data represent an enrichment for stable protein complexes, then our proposed evolutionary route appears to be more prevalent in stable complexes. In fact, most examples discussed in the text are stable complexes. They also appear to be complexes that were established very early in evolution, which is illustrated by the ages of the proteins that establish homomeric interactions and interactions between duplicates.

We have shown previously that duplication of protein interactions and of entire protein complexes is accompanied by specialization of function [9]. Inspection of the effects of duplication of homo-interactions suggests a similar outcome. In other words, the main function is established when the homomer is first formed, and then duplications will serve to specialize these functions. For example, in Figure 3a the transition from homodimer to dimers of paralogous proteins of the helix-turn-helix transcription factors results in specialization of the function of the complex, that is, distinct but overlapping specificities in DNA binding [34,35]. Other examples of functional specialization are in the ATP synthase and proteosome families, as discussed in Additional data file 1.

## Conclusion

Our investigations of protein interactions and protein complexes, as well as theoretical modeling, reveal that many protein complexes evolved by the initial establishment of self-interactions followed by duplication of these self-interacting proteins. Our study provides the first insight into the evolution of functional modularity in protein-protein interaction networks, and the origins of a large class of protein complexes.

## Materials and methods

### Datasets of protein interactions and protein complexes

Binary physical protein-protein interactions for *S. cerevisiae *[36,37], *Drosophila melanogaster *(high confidence interactions) [38] and *Caenorhabditis elegans*. [39], as well as protein complex datasets for *S. cerevisiae *[36,40,41] and complexes of known three-dimensional structure used in this study [29] are summarized in Table 1.

A non-redundant set of protein complexes of known structure, based on the PQS database as of June 2005, was prepared by considering complexes as graphs where nodes are the protein subunits (labeled by the domain architecture and chain identity) and edges are a contact between these subunits: two complexes were considered identical when they had the same subunits (same domain architectures, that is, identical or homolgous chains) and the same contact topology between subunits. Details of this procedure can be found in [42].

### Detection of gene duplication and contacts between chains

We used domain architecture as defined in the Superfamily database [43,44] to identify paralogous proteins in PINs, that is, those proteins resulting from duplication of the corresponding genes. The SUPERFAMILY database provides protein domain assignments, at the SCOP 'superfamily' level [45], for the predicted protein sequences in completed genomes. Domain assignments were generated using a curated set of profile hidden Markov models. In this work, two proteins are considered paralogous if they display the same amino- to carboxy-terminal domain architecture, ignoring gaps and tandem domain repetitions as described in [9]. Domain assignments were based on Superfamily release 1.63 [44].

In the analysis of protein complexes from PQS we considered two chains to be identical when strict sequence identity was found, and accepted gaps at the amino and carboxyl termini of the sequences. Two chains were considered homologous when they displayed the same amino- to carboxy-terminal SCOP superfamily domain architecture, and to be different when they did not satisfy any of the above criteria. We used a cut-off of five amino acids with atoms within their van der Waal's radii plus 0.5 Å for two chains to be considered in contact. The expected frequency for a given chain to form a homo- or a paralogous contact (P_h _and P_p_, respectively) within a complex was calculated by counting the number of times the given chain made one or more homo- or paralogous contacts (N_h _and N_p_, respectively) in a set resulting from 500 randomizations of that protein complex. Randomizations consisted of considering the topology of each complex fixed, and shuffling the position of each chain within the complex. The expected frequencies were estimated by P_h _= N_h_/500 and P_p _= N_p_/500.

### Network randomization

To investigate the effect of correlations in the network in terms of evolutionary relationships or topological organization, the following randomization schemes were applied.

#### Randomization by domain architecture shuffling

To test for statistical significance of the measured parameters, we performed 10,000 network randomizations, in which the topology of the network was kept constant, and the evolutionary relationships between proteins, that is, their Superfamily domain assignments, were shuffled.

#### Randomization by link shuffling

To measure the influence of local organization of network structure, link shuffling was used [46]. Repeated swapping of interaction partners among pairs of interacting proteins preserves the degree of each individual node in the network but destroys higher order topological correlations and structures such as clustering.

### Modeling of the growth of the network by gene duplication

We implemented a theoretical model of network evolution based on the concepts proposed in [11,25,26]. In this model we started with x = 340 proteins, representing the total number of 241 protein families and 29% of unassigned proteins in the Yeast dataset. We randomly introduced an interaction between any pair of proteins with a probability 0.0059 = 2/339, leading to a classic random graph with a Poissonian degree distribution and an average degree of two. The network is then allowed to grow until it reaches the same size as the Yeast network (neglecting isolated nodes generated during the simulation). The parameter δ for the probability to delete a link under duplication and α for random re-linking of a new node to older nodes in the network was chosen with the aim of obtaining realistic network features (that is, degree distribution) in the final network, that is δ = 0.9 and α = 0 or α = 0.1. For more details, see Supplementary information S2 in Additional data file 1.

### Phylogenetic profiling

We used Smith-Waterman alignments to identify orthologs of yeast genes in the genomes of 40 organisms, representing the three branches of the tree of life, and the major taxonomical groups within each of the branches. We used the Smith-Waterman implementation of the TimeLogic's DeCypher^® ^accelerated hardware. The significance of each hit is based on a PSCORE statistic where the *p *value is a real number between 0 and 1 describing the probability of a hit being random. The significance is based on the histogram fitting method and we used a cutoff of *p *< 0.01. A complete list of the organisms studied is shown in Additional data file 1 (Supplementary material S5). We considered two proteins to be orthologous if they were bidirectional best hits. The 'age' groups we can define based on available genomes are, starting from the most recent, '*S. cerevisiae *specific'; 'Saccharomyceta'; 'Fungi'; 'Fungi/Metazoa'; 'Fungi/Metazoa/Amoebozoa'; 'Eukaryota'; 'Eukaryota+Archaea' or 'Eukaryota+Bacteria'; 'universal'. The eukaryotic tree used as reference is that in [47].

## Additional data files

The following additional data are available with the online version of this paper. Additional data file 1 contains additional figures as well as raw data for the plots in Figures 4 and 5. The data used and results from this study can be accessed from the companion website [48].

## Supplementary Material

Additional data file 1 Additional figures and raw data for the plots in Figures 4 and 5 Click here for file

## Figures and Tables

**Table 1:** Data sets investigated in this study

			Pairwise interactions (%)						
				
Dataset	PPIs/Complexes	No. of proteins	HD	PD	F(HD)	F(PD)	F(HI)	F(PI)	Description
**Pairwise interactions**									

Yeast [36]	1,011	753	1.9	13.4					Manual curation of small scale data (does not include yeast two hybrid data)
Yeast-large [37]	15,393	4,741	1.8	6.2					Compilation of small- and large-scale data
Worm [39]	2,422	1,726	1.6	3.3					High-throughput (yeast two-hybrid)
Fly [38]	3,384	2,877	2.9	9.1					High-throughput (yeast two-hybrid)
				
**Complexes**									
MIPS [36]	216	1,185			32	27			Manual curation
TAP [40]	589	1,474			31	30			High-throughput tagging and mass spectometry
HMS-PCI [41]	741	1,758			33	27			High-throughput tagging and mass spectometry
PQS [29]	2509	3,124					85	11	Three-dimensional structures of protein complexes

PPIs/Complexes are the number of protein-protein interactions and protein complexes (for complexes) in the data sets, respectively. HD, homodimers; PD, dimers of paralogous proteins; F(HD) and F(PD) represent the frequency at which the complexes contain homodimers or dimers of paralogous proteins in any of the two *S. cerevisiae *protein interaction datasets. These numbers were obtained by computationally superimposing the PINs onto the complex data and are significantly higher than expected by chance at *p *< 10^-4 ^in all cases. F(HI) and F(PI) are the frequency of complexes with homomeric or paralogous interactions, respectively.

**Table 2:** Evolutionary origin of dimers of paralogues

	P(HD)	P(PD)	P(HD|PD)	P(PD|HD)
Yeast	0.034 ± 0.006	0.134 ± 0.011	0.043 ± 0.006	0.17 ± 0.012
Yeast-large	0.027 ± 0.001	0.062 ± 0.002	0.203 ± 0.003	0.466 ± 0.004
Fly	0.047 ± 0.004	0.091 ± 0.006	0.082 ± 0.006	0.257 ± 0.008
Worm	0.031 ± 0.003	0.033 ± 0.003	0.355 ± 0.008	0.379 ± 0.008

Dimers of paralogues are frequently also homodimers. HD, homodimer; PD, dimer of paralogues. P(HD|PD) should be read as the conditional probability of a polypeptide forming a homodimer given that it also participates in forming a dimer of paralogues. The standard deviations for each probability are calculated from √(p(1 - p)/n) where p is the estimated probability and n the number of observations. The enrichment observed with the conditional probabilities is significant for all interaction datasets except the small yeast network.

**Table 3:** Conservation of yeast protein interactions

	P(Fly)	P(Fly|HPD)	P(Worm)	P(worm|HPD)
Yeast	0.020 (10/409)	0.051 (4/79)	0.004 (2/457)	0.027 (2/75)
Yeast-large	0.009 (56/6113)	0.061 (34/559)	0.001 (3/5823)	0.005 (3/547)

We consider a protein interaction in yeast to be conserved in another organism if both interacting proteins in yeast have orthologs in the other organism, and the orthologous proteins interact. P(Fly) and P(Worm), probability of a protein interaction in a *S. cerevisiae *PIN to be conserved in Fly and Worm, respectively. P(Fly|HPD) and P(Worm|HPD), probability of a protein interaction in a *S. cerevisiae *PIN to be conserved in fly and worm given that it is a homomeric interaction or an interaction between paralogous proteins.
