# Supplementary material for: Evolution of protein complexes by duplication of homomeric interactions
Source: Genome Biol. 2007 Apr 5;8(4):R51. doi: 10.1186/gb-2007-8-4-r51 (PMC1895999; doi:10.1186/gb-2007-8-4-r51)
Supplement: Additional data file 1 — Additional figures and raw data for the plots in Figures 4 and 5 [file gb-2007-8-4-r51-S1.pdf]

Supplementary material for

Pereira-Leal, JB, Levy, ED, Kamp, C & Teichmann, SA

"Evolution of protein complexes by duplication of homomeric interactions"

1. Protein complexes and clustering of the network 2

2. Modelling network growth by duplication-divergence 2

3. Duplication does not affect clustering 4

4. Duplication of subunits in protein complexes 7

5. Phylogenetic profiling 8

6. Protein complex size and duplicated subunits 9

1. Protein complexes and clustering of the network

We use the clustering coefficient as a measure of modularity of the network. It is a convenient measure that can be used for all protein-protein interaction (PPI) datasets which we consider in this study. However, the biological significance of the clustering coefficient is not clear. In order to obtain insight into the biological properties that are reflected in the clustering coefficient, we investigated the correlation with protein complexes. **Tables S3.1** and **S3.2** contain the probabilities for a clustered protein to be part of a complex and vice-versa, for a clustering coefficient of at least 0.6, as an example.

Table S1.1 – Probability of a protein that is part of a complex to have a clustering coefficient in the network equal to or larger than 0.6.

	**Yeast**		**Yeast -large**	
	**$P(C\geq 0.6)$**	**$P(C\geq 0.6 \mid comp)$**	**$P(C\geq 0.6)$**	**$P(C\geq 0.6 \mid comp)$**
**MIPS**	0.43	0.45	0.06	0.09
**TAP**	0.43	0.52	0.06	0.07
**HMS-PCI**	0.43	0.52	0.06	0.07

Table S1.2 – Probability of a protein to be part of a complex if its clustering coefficient in the network is equal to or larger than 0.6.

	**Yeast**		**Yeast -large**	
	**$P(comp)$**	**$P(comp \mid C\geq 0.6)$**	**$P(comp)$**	**$P(comp \mid C\geq 0.6)$**
**MIPS**	0.65	0.68	0.27	0.38
**TAP**	0.605	0.73	0.38	0.43
**HMS-PCI**	0.512	0.54	0.41	0.19

2. Modelling network growth by duplication-divergence

We implemented a theoretical model of network evolution based on the concepts proposed in [11, 25, 26]. In this model we start with x=340 proteins, representing the total number of 241 protein families and a fraction of 29% of unassigned proteins in the

Yeast dataset. We randomly introduce an interaction between any pair of proteins with a probability $0.0059 = \frac{2}{(340-1)}$, leading to a classical random graph with a Poissonian degree distribution and an average degree of 2. Then we grow the network by iterating the following steps (*cf.* figure below).

1. Choose a random node.
2. Copy the random node and introduce a link to any of the original node's neighbours with probability 1-δ=0.1.
3. Introduce a link from the new node to any of the older nodes in the network with probability α/(no. of nodes in the network), α=0, α=0.1.
4. Discard the new node if it has no interaction partners.

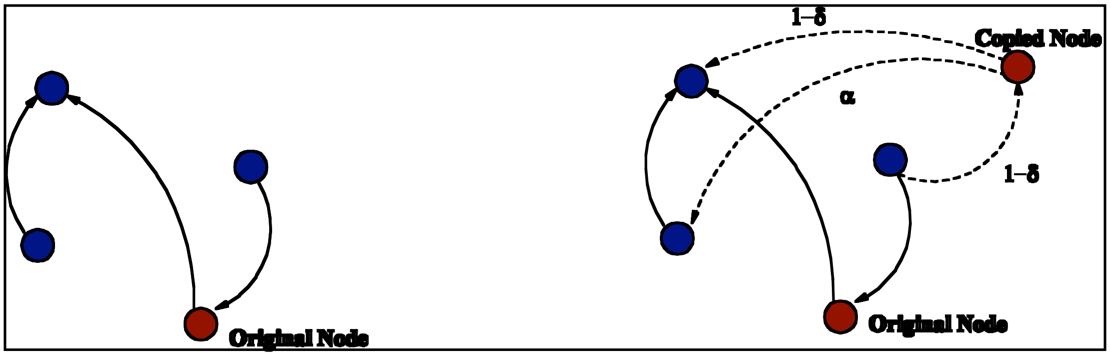

The network is then allowed to grow until it reaches the same number of 753 proteins as observed in the Yeast dataset network. The parameter δ for the probability to delete a link under duplication and α for random re-linking of a new node to older nodes in the network has been chosen with the aim of obtaining realistic network features (*i.e.* degree distribution) in the final network, that is δ=0.9 and α=0 or α=0.1. The duplication process in this algorithm slowly converges to a scale free, though not stationary, distribution. Its detailed behaviour is still a topic of current research[1].

3. Duplication does not affect clustering

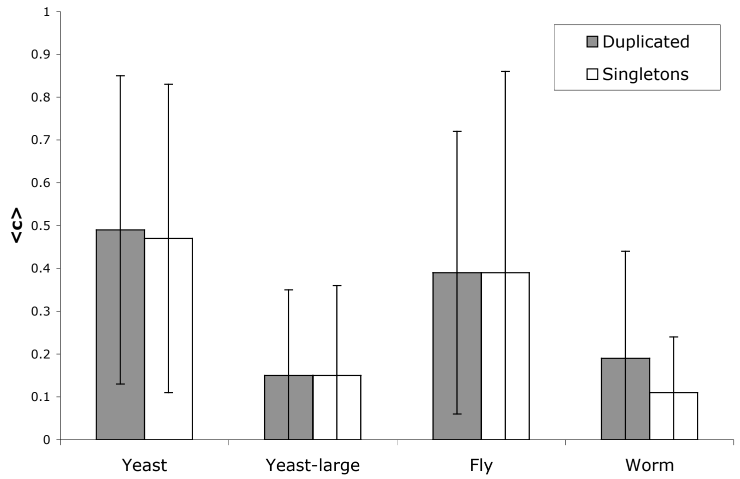

Figures S3.1 – Duplication does not increase the clustering coefficient of a protein. In this figure we plot the average clustering coefficient of proteins with and without duplicates in the protein interaction network. Error bars correspond to the standard deviation.

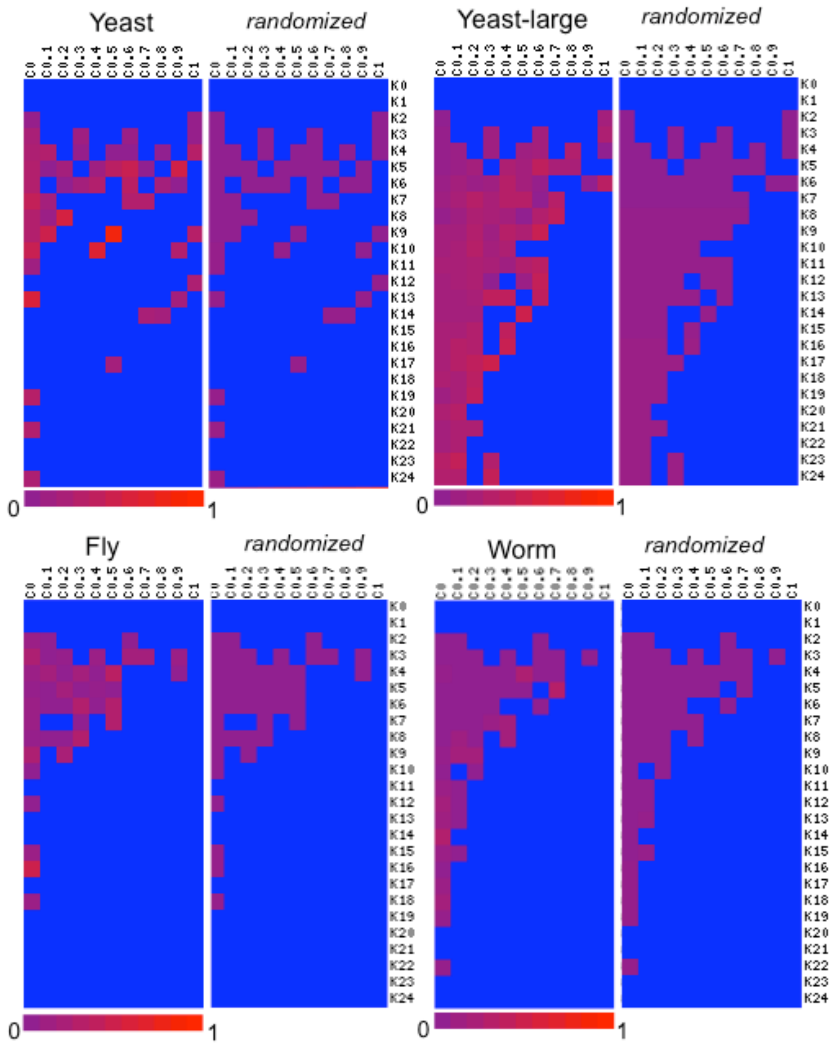

Figure S3.2 – Higher clustering coefficients are not related to duplication of a protein's binding partners. Each cell in the matrix represents the average frequency of duplication amongst the binding partners of a protein (see scale below the matrix for each dataset). The X-axis is the clustering coefficient; the Y-axis is the degree k of the proteins. Absent data points are represented in blue – these correspond to specific combinations of connectivity (side label) and clustering coefficient (top label) that are not observed in the data set. For each data set a matrix representing the average 10,000 of randomized networks is shown for comparison. Randomization involved shuffling the evolutionary relationships between proteins while keeping the topology constant. If greater clustering coefficients were linked to duplication of binding partners, then an increased in the intensity of red would be seen from left to right in the matrix.

Table S3.3 – Effect of homo- and paralogous interaction on the global clustering levels of the network. *C* is the initial clustering coefficient of the network. $C_{NoPD/HD}$ is the clustering coefficient of the network after the removal of all interactions between identical and homologous proteins. C_{Random} corresponds to the average ± standard deviation of a set of 1000 experiments in which an equivalent number of protein interactions is removed from the network and the global clustering levels are measured. Z represents the z-score, *i.e.* the number of standard deviations the $C_{NoPD/HD}$ is from the C_{Random}, and shows that the reduction in global clustering coefficient produced by removal of HD/PD is highly significant.

	**C**	$C_{NoPD/HD}$	C_{Random}	***z***	
Yeast	0.498	0.457	0.495±0.004	-9.5	p<0.001
Yeast-Large	0.132	0.113	0.132±0.0003	-63.3	p<0.001
Fly	0.050	0.046	0.050±0.0006	-6.6	p<0.001
Worm	0.075	0.070	0.075±0.0004	-12.5	p<0.001

4. Duplication of subunits in protein complexes

Table S4.1. Data for Figure 4B. - All complexes

Size	0	1	2	3	4	5	6	7	8	9	10	11	12	13	14	>=15
complexes containing paralogues	0	0	53	49	58	4	20	0	13	2	1	1	8	0	0	9
complexes not containing paralogues	0	0	977	212	575	22	247	14	111	9	19	3	55	1	9	37
% complexes containing paralogues	0.0	0.0	5.1	18.8	9.2	15.4	7.5	0.0	10.5	18.2	5.0	25.0	12.7	0.0	0.0	19.6

Table S4.1. Data for Figure 4B. - All non-homomeric complexes

Size	0	1	2	3	4	5	6	7	8	9	10	11	12	13	14	>=15
complexes containing paralogues	0	0	53	49	58	4	20	0	13	2	1	1	8	0	0	9
complexes not containing paralogues	0	0	163	40	141	7	69	5	34	7	3	0	19	1	0	24
% complexes containing paralogues	0.0	0.0	24.5	55.1	29.1	36.4	22.5	0.0	27.7	22.2	25.0	100.0	29.6	0.0	0.0	27.3

Table S4.3. Raw Data for Figure 5A

a) Frequencies

	0-0.1	0.1-0.2	0.2-0.3	0.3-0.4	0.4-0.5	0.5-0.6	0.6-0.7	0.7-0.8	0.8-0.9	0.9-1	1
Identical	0.00	0.12	0.20	0.32	0.24	0.44	0.47	0.53	0.73	0.89	1.00
Paralogous	0.00	NaN	1.00	0.97	1.00	0.88	0.96	0.96	0.98	1.00	1.00
Different	0.00	NaN	0.33	0.41	0.42	0.59	0.82	0.96	0.90	0.98	0.99

b) Corresponding absolute numbers

	0-0.1	0.1-0.2	0.2-0.3	0.3-0.4	0.4-0.5	0.5-0.6	0.6-0.7	0.7-0.8	0.8-0.9	0.9-1	1
Identical	0	20	33	53	24	280	216	128	213	253	7042
Paralogous	0	0	6	34	18	89	118	149	165	159	496
Different	5	0	3	7	8	42	76	266	226	471	1632

Table S4.4 Raw Data for Figure 5C

a) Frequencies

	**0-0.1**	**0.1-0.2**	**0.2-0.3**	**0.3-0.4**	**0.4-0.5**	**0.5-0.6**	**0.6-0.7**	**0.7-0.8**	**0.8-0.9**	**0.9-1**	**1**
Identical	0	0.12	0.2	0.32	0.24	0.44	0.47	0.53	0.73	0.89	1
Paralogous	0	NaN	1	0.97	1	0.88	0.96	0.96	0.98	1	1

b) Corresponding absolute numbers

	0-0.1	0.1-0.2	0.2-0.3	0.3-0.4	0.4-0.5	0.5-0.6	0.6-0.7	0.7-0.8	0.8-0.9	0.9-1	1
Identical	0	20	33	53	24	280	216	128	213	253	7042
Paralogous	0	0	6	34	18	89	118	149	165	159	496

5. Phylogenetic profiling

We chose representatives from the three domains of life in order to determine the phylogenetic extent of orthologs of all yeast genes (see below).

**Species**	**Partial taxonomy**
Oryza sativa	Eukaryota; Viridiplantae; Streptophyta
Arabidopsis thaliana	Eukaryota; Viridiplantae; Streptophyta
Chlamydomonas reinhardtii	Eukaryota; Viridiplantae; Chlorophyta
Dictyostelium discoideum	Eukaryota; Mycetozoa; Dictyosteliida
Caenorhabditis elegans	Eukaryota; Metazoa; Nematoda
Mus musculus	Eukaryota; Metazoa; Chordata
Homo sapiens	Eukaryota; Metazoa; Chordata
Danio rerio	Eukaryota; Metazoa; Chordata
Fugu rubripes	Eukaryota; Metazoa; Chordata
Anopheles gambiae	Eukaryota; Metazoa; Arthropoda
Drosophila melanogaster	Eukaryota; Metazoa; Arthropoda
Ustilago maydis	Eukaryota; Fungi; Basidiomycota; Ustilaginomycetes
Schizosaccharomyces pombe	Eukaryota; Fungi; Ascomycota; Schizosaccharomycetes
Saccharomyces paradoxus	Eukaryota; Fungi; Ascomycota; Saccharomycotina
Saccharomyces mikatae	Eukaryota; Fungi; Ascomycota; Saccharomycotina
Saccharomyces kudriavzevii	Eukaryota; Fungi; Ascomycota; Saccharomycotina
Saccharomyces castellii	Eukaryota; Fungi; Ascomycota; Saccharomycotina
Saccharomyces bayanus	Eukaryota; Fungi; Ascomycota; Saccharomycotina
Saccharomyces kluyveri	Eukaryota; Fungi; Ascomycota; Saccharomycotina
Aspergillus nidulans	Eukaryota; Fungi; Ascomycota; Pezizomycotina
Trypanosoma brucei	Eukaryota; Euglenozoa; Kinetoplastida
Plasmodium falciparum	Eukaryota; Alveolata; Apicomplexa
Vibrio cholerae	Bacteria; Proteobacteria; Gammaproteobacteria
Pseudomonas putida KT2440	Bacteria; Proteobacteria; Gammaproteobacteria

Haemophilus influenzae Rd KW20	Bacteria; Proteobacteria; Gammaproteobacteria
Yersinia pestis CO92	Bacteria; Proteobacteria; Gammaproteobacteria
Escherichia coli K12	Bacteria; Proteobacteria; Gammaproteobacteria
Buchnera aphidicola Bp	Bacteria; Proteobacteria; Gammaproteobacteria
Mycoplasma genitalium G-37	Bacteria; Firmicutes; Mollicutes
Clostridium acetobutylicum ATCC 824	Bacteria; Firmicutes; Clostridia
Clostridium tetani E88	Bacteria; Firmicutes; Clostridia
Bacillus subtilis ssp. subtilis 168	Bacteria; Firmicutes; Bacillales
Thermus thermophilus HB27	Bacteria; Deinococcus-Thermus
Synechococcus sp. WH 8102	Bacteria; Cyanobacteria; Chroococcales
Mycobacterium tuberculosis H37Rv	Bacteria; Actinobacteria; Actinobacteridae
Nanoarchaeum equitans Kin4-M	Archaea; Nanoarchaeota; Nanoarchaeum
Thermoplasma acidophilum DSM 1728	Archaea; Euryarchaeota; Thermoplasmatasma
Pyrococcus furiosus DSM 3638	Archaea; Euryarchaeota; Thermococci
Methanocaldococcus jannaschii DSM 2661	Archaea; Euryarchaeota; Methanococci
Aeropyrum pernix K1	Archaea; Crenarchaeota; Thermoprotei

6. Protein complex size and duplicated subunits

We investigated whether protein complexes that have duplicated subunits are different from other protein complexes. There is a distinction between the two groups in terms of the number of different proteins in the complexes. This difference is statistically significant, as can be seen in **Table S6.1** and **Figure S6.2.**

Table S6.1. Average number of distinct proteins in yeast protein complexes. Significance was assessed by a two-tailed t-test at 5% confidence.

	Average number of proteins in complexes		
	**with duplicates**	**without duplicates**	**significance**
**MIPS**	9.7	4.1	$P<2.6x10^{-5}$
**TAP**	11.9	3.9	$P<4.6x10^{-38}$
**HMS-PCI**	11.1	3.4	$P<2.4x10^{-29}$

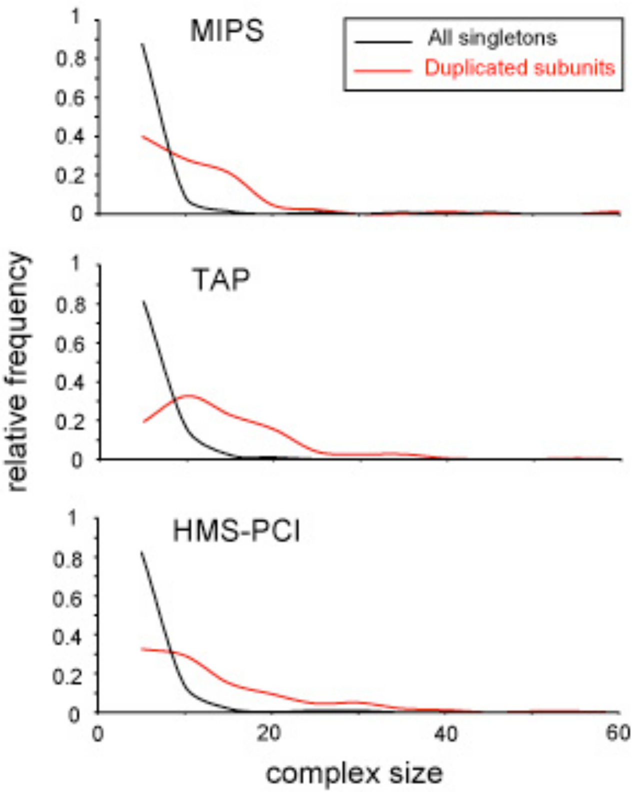

Figure S6.2. Distribution of protein complex sizes in three protein complex datasets in yeast. Complex size refers to the number of distinct proteins. Complex sizes are represented in bins of size 5. The complexes without paralogous proteins (all singletons) tend to be smaller than complexes containing duplicates, shown in red.

7. Examples of functional specialization

One example of functional specialization of a complex by duplication is the 20S particle of the proteasome that in Archaea is coded by two genes, whereas in Eukaryota, it consists of up to fourteen different paralogues. In both organisms the 20S particle is composed of four stacked heptameric rings. In the Archaea, two paralogous α and β subunits form the two outer and central rings respectively, with extensive homomeric contacts[53]. In eukaryotes, extensive gene duplication results in the seven α and β subunits being coded by paralogous proteins, and paralogous contacts predominate[54]. The diverse subunits in eukaryotes allow specialized catalytic functions[55], *i.e.*, sub-functionalization at the level of the protein complex[56]. These duplications are associated with specialized catalytic functions.

The evolution of the F1/Fo family of proton-transporting membrane complexes is another example of how gene duplication can generate functional versatility. The ancestral complex is thought to have consisted of six catalytic subunits in the F1 part,

and 12 ‘c’ subunits in the Fo part which pumped protons. Duplication of the catalytic subunit with subsequent inactivation of the duplicate created the current function of the ATP synthase where protons flow down the gradient. (The hetero-hexameric F1 is shown in **Figure 4B,** left). A second reversal in the direction of proton flow was accomplished in evolution by duplication followed by gene fusion of the paralogues of the ‘c’ subunit, creating a proton/ATP ratio that is compatible with the pumping of protons, as observed in the vacuolar ATPase[57].
